# Predicted Vancomycin Dosage Requirement in Patients With Hematological Malignancies and Dosage Dynamic Adjustment

**DOI:** 10.3389/fphar.2022.890748

**Published:** 2022-06-06

**Authors:** Xiangqing Song, Yi Wu

**Affiliations:** Department of Pharmacy, Hunan Cancer Hospital/The Affiliated Cancer Hospital of Xiangya School of Medicine, Central South University, Changsha, China

**Keywords:** vancomycin, *Staphylococcus* spp, hematological malignancies, pharmacokinetic/pharmacodynamic, Monte Carlo simulations, dosage dynamic adjustment

## Abstract

**Purpose:** The purpose of this study was 1) to predict the requisite vancomycin daily dose (*D*_*van*_) used in the target patients suffering from both bacterial infection and hematological malignancies and 2) to construct a vancomycin-dose-graphical tool to assist clinicians to develop vancomycin dosing regimens and further 3) to establish a programming process for vancomycin dynamic dosage adjustment to help clinicians to adjust vancomycin dosing regimens according to physiological and pathogenic factors of the target patients.

**Methods:** The *D*_*van*_ model associated with microbial susceptibility, vancomycin pharmacokinetics, and dosing parameters was established, and the *D*_*van*_ was estimated based on the established *D*_*van*_ model and using Monte Carlo simulations. *D*_*van*_ achieving 90% of probability of target attainment (PTA) for bacterial isolate or cumulative fractions of response (CFR) for the bacterial population at a ratio of daily area under the curve (AUC_24_) to the minimum inhibitory concentration (MIC) [i.e., AUC_24_/MIC] of 400–600 was considered sufficient to treat infection occurring in the target patients. On the basis of the predicted *D*_*van*_, the physiological states of patients, and the pathogenic variables of infection, a vancomycin-dose-graphical tool for the target patients and a programming process for vancomycin dynamic dosage adjustment were constructed.

**Results:** This study predicted the requisite *D*_*van*_ used in patients suffering from both bacterial infection and hematological malignancies and constructed a vancomycin-dose-graphical tool for the target patients, at different physiological states and pathogenic variables, to formulate vancomycin dosing regimens. Also, this study established and expounded the formulation process of vancomycin dosage dynamic adjustment according to fluctuant renal function of the target patients.

**Conclusion:** With the tools, the required *D*_*van*_ or vancomycin dosing regimens for the target patients, at different physiological states and pathogenic variables, can be readily known, whether or not vancomycin dynamic dosage adjustment is required.

## Introduction

As an antibiotic widely used for infections due to Gram-positive bacteria, especially those with antibiotic resistance, vancomycin (VAN) is often the last line of defense no matter whether these infections occur in patients with or without cancer.

However, when VAN is used in patients with cancer, serious concerns in toxicity, pathogen resistance, and therapeutic failure resulted from inappropriate VAN dosage should be of particular consideration, since compared with general groups, patients with cancer often show distorted PK variability for VAN (Chang et al., 1994; Le Normand et al., 1994; Chang, 1995; Krivoy et al., 1998; Sadoh et al., 2010), such as significantly elevated VAN clearance (*CL*_van_) and distribution volume (*V*_d_) (Le Normand et al., 1994; Al-Kofide et al., 2010; Sadoh et al., 2010; Curth et al., 2015; Izumisawa et al., 2019; Alqahtani et al., 2020; Zhang and Wang, 2020). This phenomenon, known as augmented renal clearance, may make VAN exposure insufficient when VAN is at conventional dosage. This outcome results in potentially increased resistance and therapy failure, which are associated with infection-related morbidity and mortality. Unfortunately, this phenomenon and causal consequences are infrequently considered in most contemporary dosing regimens, although systemic inflammatory response syndrome or the malignant state in patients with cancer may be covariates of VAN PK variability. The 2020 VAN therapeutic guideline issued by the American Society of Health-System Pharmacists recommends VAN dosage for infected adults and pediatric patients, including those special groups with obesity or dialysis, but it does not include recommendations for VAN dosage used in patients with cancer (Rybak et al., 2020b).

Hence, a challenge in regulating the use of VAN in the management of infections occurring in these patients appears. Moreover, another challenge encountered clinically is that during VAN treatment, some clinicians often ignore VAN dynamic dosage adjustment based on the physiological variables (e.g., fluctuant renal function) of patients. However, the outcome due to this negligence is important for patients with acute renal impairment or recovery, as VAN is primarily excreted by the kidney and has a narrow therapeutic window. Fluctuations in renal function can directly affect excretion and exposure of VAN and further lead to variation of VAN in efficacy and toxicity. These challenges speak about the criticality and significance of constructing predicted VAN dosage for cancer patients and of reconsidering the habitual “one dose fits all cases” approach to ensure satisfactory treatment.

To our knowledge, currently, no approach can determine accurate VAN dosage for patients. The PK/PD method, in which the PK/PD index of antibiotics must have a strong correlation with clinical and microbiological outcomes, to optimize exposure to improve efficacy has shown the feasibility of such integration in many antibiotics in OPTAMA studies based on Monte Carlo simulations (MCSs) (Kuti and Nicolau, 2005), thus providing an explorable approach for other antibiotics to develop the dosing schemes and predict the outcomes. Likewise, this method is suitable for VAN because AUC_24_/MIC, a PK/PD index for VAN, has been proved to be strongly correlated with VAN response (Rybak et al., 2020b). To our knowledge, however, it has been little implemented in optimizing VAN dosage used in infected patients with hematological malignancies, with only two publications focusing on such a topic until recently (de Gatta et al., 2009; Alqahtani et al., 2020). Sufficient data are therefore still lacking. Moreover, it is believed that these studies may be more indicative and targeted if they had considered more specific MICs, detailed renal function grading, and definite dosing regimens.

These considerations prompt us to re-construct optimal VAN dosage for the target patients suffering from both bacterial infection and hematological malignancies, especially for those with fluctuant renal function. Therefore, the present study aims at 1) predicting the requisite VAN daily dose (*D*_van_) for the target patients, 2) constructing a VAN-dose-graphical tool to assist clinicians to formulate VAN dosing regimens based on the predicted *D*_van_, the physiological states of patients, and the pathogenic variables of infection, and further 3) establishing a programming process for VAN dynamic dosage adjustment to help prescribers to adjust VAN dosing regimens according to fluctuant physiological and pathogenic factors. It is expected that the required VAN dosage for the target patients can be readily known, and the findings can substantially provide help in the presence of lacking in therapeutic drug monitoring.

## Materials and Methods

### Study Design

The *D*_van_ model associated with microbial susceptibility, VAN PK, and dosing parameters was established. VAN population PK parameters derived from patients with hematological malignancies, microbial susceptibility derived from the Antimicrobial Testing Leadership and Surveillance (ATLAS) database and emulation dosing parameters derived from medication practice, were incorporated as the variables into the *D*_van_ model to estimate the *D*_van_ based on using MCSs. *D*_van_ achieving 90% of probability of target attainment (PTA) for bacterial isolate or cumulative fractions of response (CFR) for the bacterial population at an AUC_24_/MIC ratio of 400–600 was considered sufficient to treat infection occurring in the target patients. On the basis of the predicted *D*_van_, the physiological states of patients, and the pathogenic variables of infection, a VAN-dose-graphical tool for the target patients and a programming process for VAN dynamic dosage adjustment were constructed.

### Vancomycin PK/PD “Efficacy” Target and the Initial *D*_van_ Model

In the 2020 VAN therapeutic guideline (Rybak et al., 2020b), when VAN is used to treat bacterial infection, an AUC_24_/MIC ratio of 400–600 (assuming an MIC of 1 mg/L determined by broth microdilution) as the primary PK/PD “efficacy” target is recommended considering the balance of nephrotoxicity and efficacy of VAN, and trough-only monitoring, with a target of 15–20 mg/L, is no longer recommended. Thus, an AUC_24_/MIC ratio of 400–600 was used as the optimal VAN PK/PD “efficacy” target in this study. Of note, the AUC_24_/MIC ratio of 400–600 refers to the total AUC_24_/MIC values unless the ratio is designated as *f*AUC_24_/MIC (*f* is the fraction of the unbound drug) since the total and free AUC_24_/MIC (i.e., 50% protein binding × AUC_24_/MIC) for VAN have been interchangeably reported (Rybak et al., 2009). In the administration mode of using intermittent infusion, the calculation model of the AUC_24_/MIC ratio at a steady state is derived from deduction (see Supplementary Appendix S1: Derivation of AUC_24_/MIC) as follows:

$$\text{A}\text{U}{\text{C}}_{24}/\text{M}\text{I}\text{C}=\frac{\frac{24v}{\tau \cdot \left(e^{CL_{van}/V_{d}\cdot \tau }-e^{CL_{van}/V_{d}\cdot t_{\inf}}\right)}\left[\frac{t_{\inf}\left(e^{CL_{van}/V_{d}\cdot \tau }-1\right)}{CL_{van}}-\frac{{\left(e^{CL_{van}/V_{d}\cdot t_{\inf}}-1\right)}^{2}}{V_{d}}\right]}{\text{M}\text{I}\text{C}},$$ (1)

where AUC_24_ (mgh/L) is daily area under the curve, MIC (mg/L) is minimum inhibitory concentration, *CL*_van_ (L/h) is VAN clearance, *V*_*d*_ (L) is distribution volume, *t*_inf_ (h) is infusion time, *τ* (h) is dosing interval, *v* (mg/h) is the zero-order infusion rate, calculated as each dose divided by infusion time [i.e., *D*_van_/(24/τ)/*t*_inf_], and *e* is the natural constant.

Since the AUC_24_/MIC value, designated as 400–600, is a constant reflecting PK/PD “efficacy,” here we set it to “*φ*.” Then,

$$\varphi =\frac{\frac{24v}{\tau \cdot \left(e^{CL_{van}/V_{d}\cdot \tau }-e^{CL_{van}/V_{d}\cdot t_{\inf}}\right)}\left[\frac{t_{\inf}\left(e^{CL_{van}/V_{d}\cdot \tau }-1\right)}{CL_{van}}-\frac{{\left(e^{CL_{van}/V_{d}\cdot t_{\inf}}-1\right)}^{2}}{V_{d}}\right]}{\text{MIC}}.$$ (2)

Due to the relationship of *v* = *D*_*van*_/(24/τ)/*t*_inf_, the relation of AUC_24_/MIC (i.e., *φ*) and *D*_*van*_ is as follows:

$$D_{\text{v}\text{a}\text{n}}=\frac{\varphi \cdot \text{M}\text{I}\text{C}\cdot \left(e^{CL_{\text{v}\text{a}\text{n}}/V_{d}\cdot \tau }-e^{CL_{\text{v}\text{a}\text{n}}/V_{d}\cdot t_{\inf}}\right)}{\frac{\left(e^{CL_{\text{v}\text{a}\text{n}}/V_{d}\cdot \tau }-1\right)}{CL_{\text{v}\text{a}\text{n}}}-\frac{{\left(e^{CL_{\text{v}\text{a}\text{n}}/V_{d}\cdot t_{\inf}}-1\right)}^{2}}{t_{\inf}\cdot V_{d}}}.$$ (3)

### Vancomycin Population PK Parameters and the Final *D*_van_ Model

According to Eq. 3, determination of the *D*_*van*_ value requires the determination of PK parameters (i.e., *CL*_*van*_ and *V*_*d*_). Ideally, the individualized PK parameters of the target patients should be used because they are the most representative ones, especially in individualized dosing. However, these data are difficult to be obtained. Thus, the population PK parameters documented (preferably from the target population) were used as surrogates in the present study. The VAN population PK parameters, i.e., *CL*_*van*_ (L/h) = 1.08 × [creatinine clearance (*CL*_*cr*(Cockcroft and Gault)_, in L/h)] and *V*_*d*_ (L) = 0.98 × total body weight (TBW, in kg), which were constructed based on the patients with hematological malignancies by Buelga et al. (2005), were chosen for our analysis. Regarding the reasons for the choice of these data, since 1) the a *priori* performance of these models was evaluated in another 59 patients and clinical suitability was confirmed, 2) these models were accurate, with more than 33% of the measured concentrations being within ±20% of the predicted value, and 3) the therapeutic precision is two-fold higher than that of a non-customized population model (16.1%), the corresponding standardized prediction errors included zero and a standard deviation close to unity.

Due to the relationship established previously, it is understandable that *CL*_*cr*_ and *TBW* can be used as surrogates for *CL*_*van*_ and *V*_*d*_ to be simulated and may be more popular since these data are more accessible. Therefore, a modified final *D*_*van*_ model can be expressed as follows:

$$D_{\text{v}\text{a}\text{n}}=\frac{\varphi \cdot \text{M}\text{I}\text{C}\cdot \left(e^{\frac{1.08\cdot CL_{cr}}{0.98\cdot TBW}\cdot \tau }-e^{\frac{1.08\cdot CL_{cr}}{0.98\cdot TBW}\cdot t_{\inf}}\right)}{\frac{\left(e^{\frac{1.08\cdot CL_{cr}}{0.98\cdot TBW}\cdot \tau }-1\right)}{1.08\cdot CL_{cr}}-\frac{{\left(e^{\frac{1.08\cdot CL_{cr}}{0.98\cdot TBW}\cdot t_{\inf}}-1\right)}^{2}}{t_{\inf}\cdot 0.98\cdot TBW}}.$$ (4)

### Simulated TBW, *CL*_*cr*_, and Pathogen MIC

**FIGURE 1:**
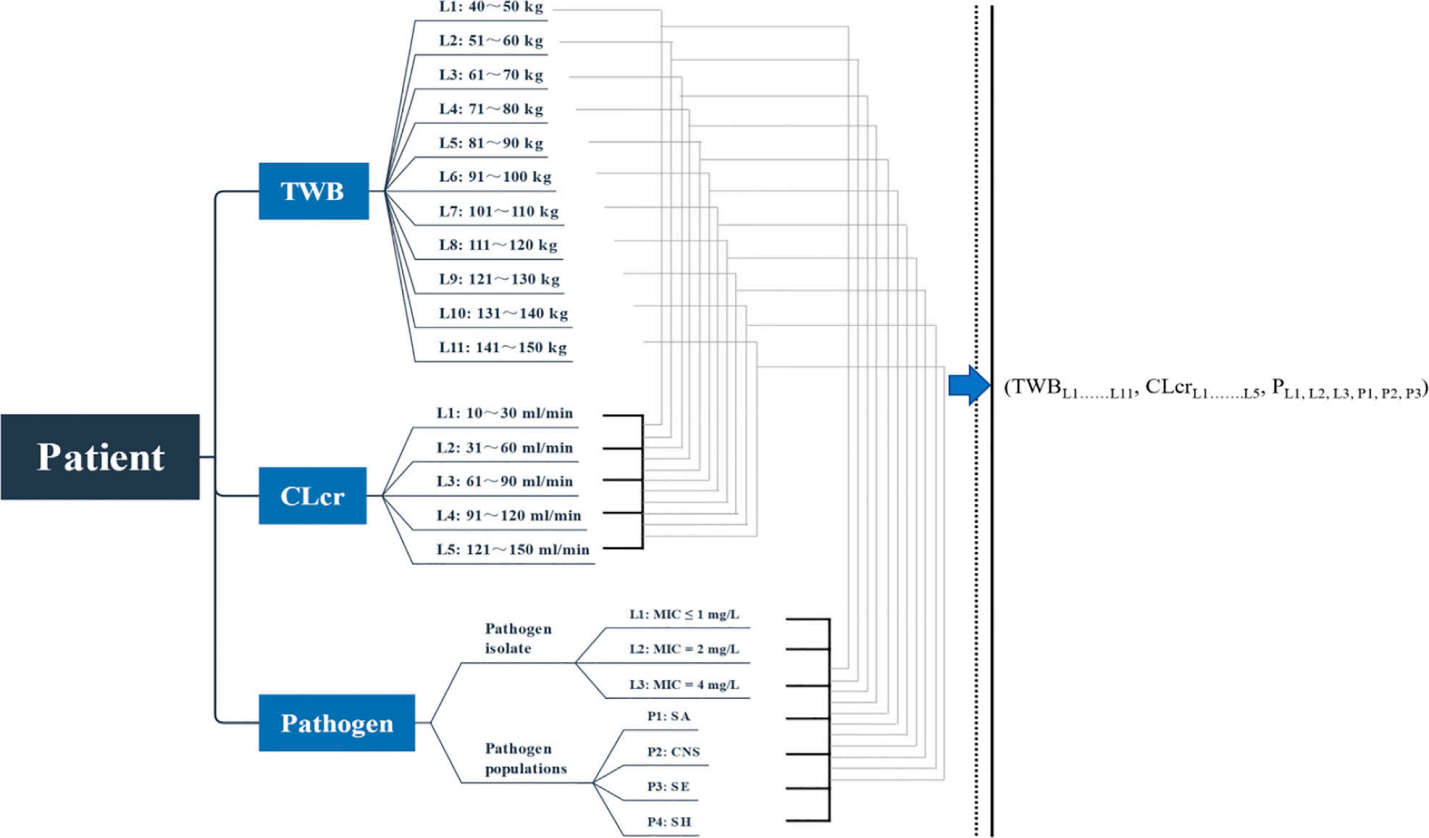
Simulated combination states of TBW–*CL*_cr_–MIC. TBW, total body weight; *CL*_cr_, creatinine clearance; L, level; P, populations; SA, *Staphylococcus aureus*; CNS, coagulase-negative *Staphylococcus*; SE, *Staphylococcus epidermidis*; SH, *Staphylococcus haemolyticus*.

According to Eq. 4, determination of the *D*_*van*_ value requires the determination of *CL*_cr_, *TBW*, and pathogen MIC. Various stages of *CL*_*cr*_ ranging from 10–150 ml/min (with a 30 mL/min increment) and *TBW* ranging from 40–150 kg (with a 10 kg increment) were simulated. The potential pathogens selected for our analysis were *Staphylococcus* spp.: *Staphylococcus aureus* (SA) as well as coagulase-negative *Staphylococcus* (CNS), *Staphylococcus epidermidis* (SE), and *Staphylococcus haemolyticus* (SH). The MIC frequency distributions of these pathogens, included in Table 1, were derived from the Antimicrobial Testing Leadership and Surveillance (ATLAS) database in 2020 (The Micron Group, 2022). The 2020 VAN therapeutic guideline emphasizes that under most circumstances of empiric dosing, the VAN MIC should be assumed to be 1 mg/L, and it does not recommend decreasing the dose to achieve the desired AUC_24_ exposure for an MIC of even <1 mg/L (Rybak et al., 2020b). These statements imply that for MICs of even <1 mg/L, they should be identified as 1 mg/L and a VAN dosage with at least conventional levels for such MICs should be administered. An MIC of ≤1 mg/L (calculated as 1 mg/L), 2 mg/L, and 4 mg/L for the isolates and a pooled MIC for the populations were simulated, and *D*_*van*_ at any one of the physiology-infection states consisting of *TBW*–*CL*_*cr*_–MIC or pathogen species, described in Figure 1, was estimated.

**TABLE 1:** Frequency distributions of MIC for simulated pathogens.

MIC (mg/L)	Frequency (%)			
	**SA **(*N* ** = 9,554)**	**CNS **(*N* ** = 136)**	**SE **(*N* ** = 1,306)**	**SH **(*N* ** = 1,068)**
0.25	0.06	1.47	0.08	1.78
0.5	6.34	11.77	0.69	8.80
1	90.32	50.74	29.86	40.45
2	3.28	35.29	68.76	48.03
4	0	0.74	0.46	0.94
8	0	0	0	0
16	0	0	0	0
32	0	0	0.15	0

SA, *Staphylococcus aureus*; CNS, coagulase-negative *Staphylococcus*; SE, *Staphylococcus epidermidis*; SH, *Staphylococcus haemolyticus*.

### Simulated Infusion State

According to Eq. 4, determination of the *D*_*van*_ value also requires the determination of infusion parameters (i.e., *τ* and *t*_inf_). In the administration mode of using intermittent infusion, dosing for every 8–12 h is recommended in the 2020 VAN therapeutic guideline (Rybak et al., 2020b). Considering the dose-dependent nephrotoxicity of VAN, in general, per dose of ≤2,000 mg and a daily dose of ≤4,000 mg are recommended when VAN is used in adults (Lodise et al., 2008; Filippone et al., 2017; Rybak et al., 2020a; U.S. Pharmacopeia). Moreover, VAN should be diluted for ≤5 mg/mL and infused over ≥1 h or at a rate of 10–15 mg/min (≥1 h per 1,000 mg) to minimize infusion-related adverse events (Rybak et al., 2020b). Due to the limit of ≤2,000 mg per dose and of ≥1 h infusion per 1,000 mg, it is understandable that VAN with 2–3 h infusion is the most frequent, especially at a conventional dose. Therefore, infusion parameters, i.e., τ of 8 h or 12 h and *t*_inf_ of 2–3 h, were simulated in the current study.

### Monte Carlo Simulations and Predicted *D*_*van*_

The MCS method has been commonly used in antimicrobial PD studies to determine the appropriate dosage regimens for further clinical development, to help the selection of clinically relevant susceptibility breakpoints, to ascertain the effect of changing administration techniques (such as altering infusion duration) on an agent’s PD parameter of interest, or to even compare different antibiotics against selected populations of bacteria (Ambrose and Grasela, 2000; Drusano et al., 2001; Ambrose et al., 2003; Mouton et al., 2004). Regarding the principles, software application and specific implementation of the MCS method, they have been well described elsewhere (Moine et al., 2016; Song and Long, 2018; Song et al., 2021). Briefly, it is to integrate the simulated variables to estimate the predictive variable. Oracle Crystal Ball software (version 11.1.2; Decisioneering, Inc., Denver, CO, United states) was used to perform MCSs, and the physiology parameters (i.e., *CL*_*cr*_ and *TBW*), infusion parameters (i.e., *τ* and *t*_inf_), and PD parameters (i.e., MIC and AUC_24_) as the simulated variables were incorporated into the final *D*_van_ model to estimate the *D*_*van*_ value. Since the MCS method simulates thousands of patients at given simulated parameters, it is important to acknowledge assumptions made regarding the variability in these parameter estimates. Based on the characteristics of the simulated variables, custom distributions for *τ* and MIC and uniform distributions for *CL*_*cr*_, *TBW*, *φ,* and *t*_inf_ were assumed in the present study. In our analysis, a 5,000-subject MCS was performed, and the confidence interval was set to 95%.

The results of MCSs are most commonly reported as: 1) the likelihood of a dosage regimen obtaining the targeted exposure for bacterial isolate with a specific MIC, referred to as the PTA or 2) the overall probability of a dosage regimen obtaining the targeted target for a bacterial population with pooled MICs, referred to as the CFR (Mouton et al., 2005). Regimen with the highest PTA or CFR would be optimal, as they would provide the highest likelihood of obtaining the targeted exposure for bacterial isolate or population. In antibiotic treatment, CFR and PTA are often used to measure the clinical acceptability of a dosage regimen. A 90% of PTA or CFR was assumed acceptable in clinic. With MCSs, a PTA-*D*_*van*_ (for bacterial isolate or a determinate MIC) or CFR-*D*_*van*_ (for bacterial population or pooled MICs) diagram, with *D*_van_ as the abscissa and PTA or CFR as the ordinate, was obtained. The desired *D*_van_ at the designated PTA or CFR target was acquired by assigning the PTA or CFR as the designated target value. *D*_van_ that maximized the PTA or CFR of simulated patients to 90% was defined as sufficient and acceptable.

### Construction of VAN-Dose-Graphical Tool and Predicted VAN Dosage Regimens

According to the predicted *D*_van_, a VAN-dose-graphical tool with *CL*_cr_ as the abscissa, TBW as the main ordinate, and MIC or pathogen population as the secondary ordinate can be drawn. According to the predicted *D*_van_ and τ, the predicted VAN dosage regimens can be determined and expressed in the form of “*D*_van_ (mg/day) divided every *τ* hours (i.e., *D*_van_/(24/τ), q τh).”

### Vancomycin Dosage Dynamic Adjustment Monitoring

On the basis of the dynamic monitoring of physiological variables of the target patients (e.g., *CL*_*cr*_ and *TBW*) and pathogenic variables (e.g., pathogen MIC) and the established VAN-dose-graphical tool, a programming process of VAN dosage dynamic adjustment can be formulated.

## Results

### Predicted *D*_*van*_ at Various MICs and VAN-Dose-Graphical Tool for Pathogen Isolate

**FIGURE 2:**
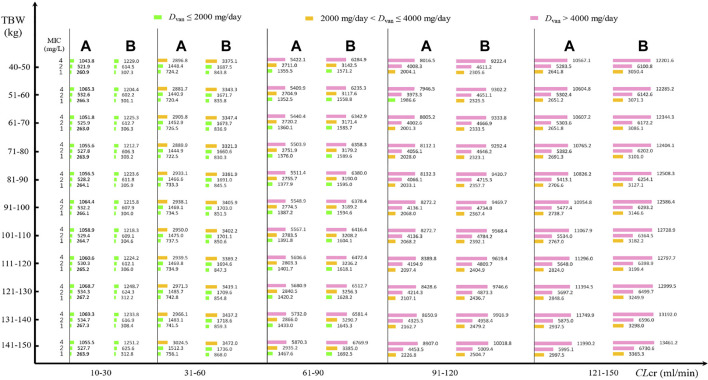
VAN daily doses for pathogen isolate with various MICs at a PTA of 90% based on MCSs (*N* = 5,000). **(A)** Daily dose divided every 8 h for different MICs; **(B)** daily dose divided every 12 h for different MICs.

Daily doses divided every 8 h or 12 h at various MICs are displayed in Figure 2. At an AUC_24_/MIC target of 400–600 and with a PTA of 90% as the clinical acceptability, VAN with approximately 270 mg/day for isolate with an MIC of 1 mg/L, 530 mg/day for isolate with an MIC of 2 mg/L, and 1,060 mg/day for isolate with an MIC of 4 mg/L in patients with *CL*_*cr*_ of 10–30 ml/min should be needed when administered every 8 h, regardless of TBW. However, approximately 740 mg/day for isolate with an MIC of 1 mg/L, 1,500 mg/day for isolate with an MIC of 2 mg/L, and 3,000 mg/day for isolate with an MIC of 4 mg/L may be requisite in patients with *CL*_cr_ of 31–60 ml/min. With an increase of *CL*_cr_ to 61–90 ml/min, VAN with approximately 1,400 mg/day for isolate with an MIC of 1 mg/L, 2,800 mg/day for isolate with an MIC of 2 mg/L, and 5,600 mg/day for isolate with an MIC of 4 mg/L should be administered. As the *CL*_*cr*_ continues to increase, however, >2,000 mg/day for isolate with an MIC of 1 mg/L, >4,000 mg/day for isolate with an MIC of 2 g/day and >8,000 mg/day for isolate with an MIC of 4 mg/L may be necessary. Unexpectedly, compared with the dosage regimen administered every 8 h, when VAN was administered every 12 h, approximately 12%–18% of these daily doses should be given additionally to obtain the desired AUC_24_/MIC and PTA targets.

Figure 2 shows the VAN-dose-graphical tool for pathogen isolate with a definite MIC. It showed the potential VAN dosage regimens at different *CL*_cr_, TBW, and pathogen MIC.

### Predicted *D*_*van*_ at Pooled MICs and Vancomycin-Dose-Graphical Tool for Pathogen Populations

**FIGURE 3:**
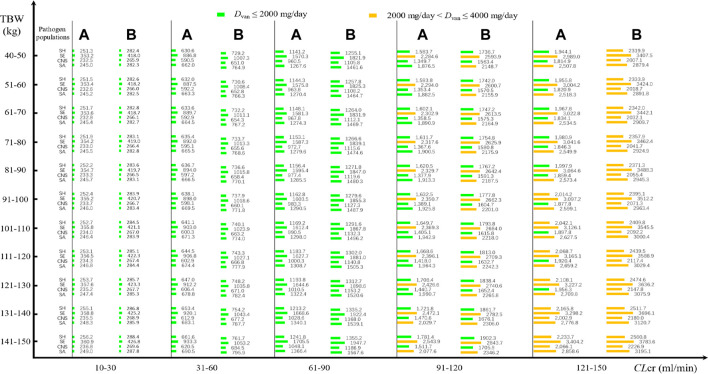
VAN daily doses for pathogen populations with a pooled MIC at a CFR of 90% based on MCSs (*N* = 5,000). **(A)** Daily dose divided every 8 h for different pathogen populations; **(B)** daily dose divided every 12 h for different pathogen populations.

**FIGURE 4:**
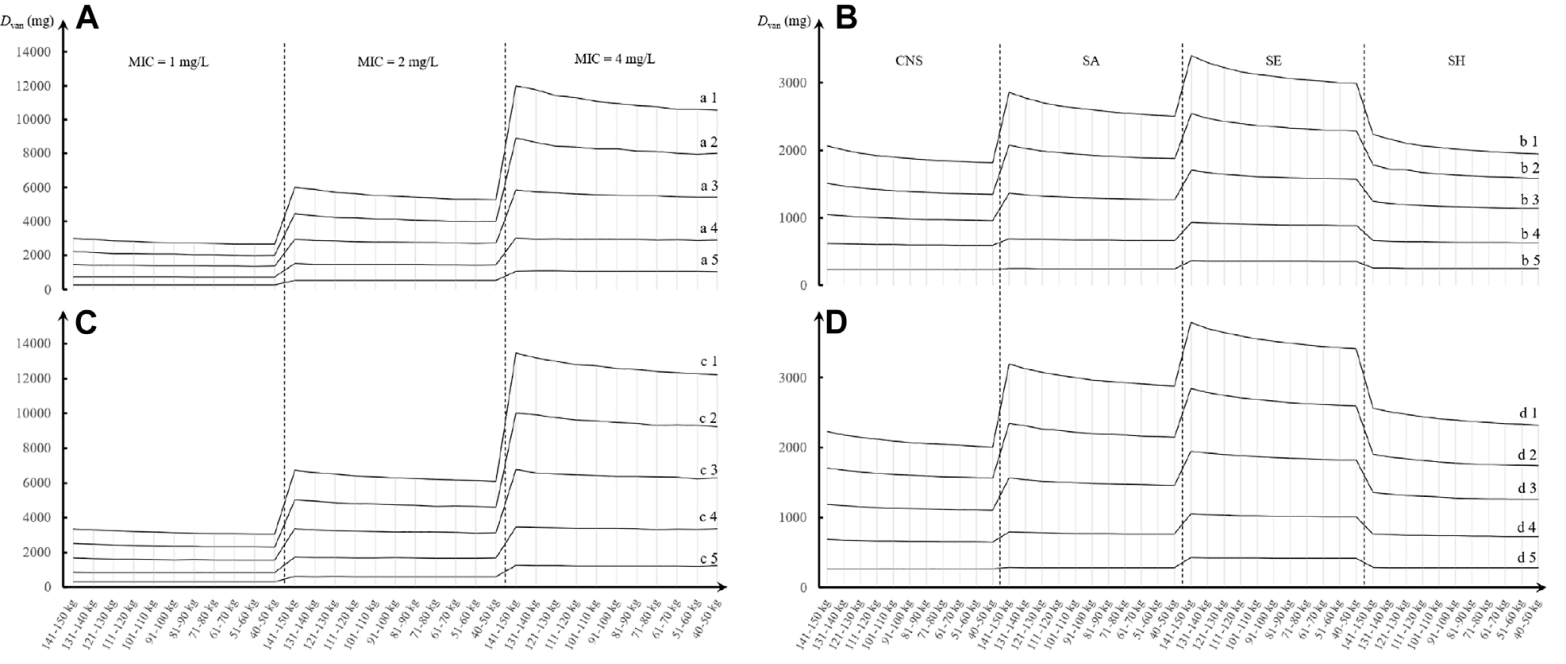
Trend chart of VAN daily dose at different TBW, *CL*_cr_, and MIC or pathogen species based on MCSs (*N* = 5,000). **(A,C)** Daily dose divided every 8 h for different MICs; **(B,D)** daily dose divided every 12 h for different pathogen populations. a1, b1, c1, and d1: *CL*_cr_ = 121–150 ml/min; a2, b2, c2, and d2: *CL*_cr_ = 91–120 ml/min; a3, b3, c3, and d3: *CL*_cr_ = 61–90 ml/min; a4, b4, c4, and d4: *CL*_cr_ = 31–60 ml/min; and a5, b5, c5, and d5: *CL*_cr_ = 10–30 ml/min. CNS, coagulase-negative *Staphylococcus*; SA, *Staphylococcus aureus*; SE, *Staphylococcus epidermidis*; SH, *Staphylococcus haemolyticus*.

Daily doses divided every 8 or 12 h at pooled MICs are presented in Figure 3. At an AUC_24_/MIC target of 400–600 and with a CFR of 90% as the clinical acceptability, VAN with approximately 250 mg/day for current SA, CNS, and SH populations and 360 mg/day for current SE populations may be adequate in patients with *CL*_*cr*_ of 10–30 ml/min when administered every 8 h, regardless of *TBW*. However, approximately 670, 600, 900, and 640 mg/day for current SA, CNS, SE, and SH populations, respectively, in patients with *CL*_*cr*_ of 31–60 ml/min, and 1,300, 1,000, 1,600, and 1,200 mg/day for these populations in patients with *CL*_cr_ of 61–90 ml/min should be administered to reach the desired AUC_24_/MIC and CFR targets. As the *CL*_*cr*_ increases to 91–120 ml/min, approximately 2,000, 1,400, 2,400, and 1,700 mg/day for current SA, CNS, SE, and SH populations, respectively, may be requisite for attaining the desired AUC_24_/MIC and CFR targets. However, approximately 2,000 mg/day for SH and CNS populations, 3,100 mg/day for SE populations, and 2,600 mg/day for SA populations may be necessary for these targets when patients have *CL*_*cr*_ of 121–150 ml/min. Interestingly, compared with the dosage regimen administered every 8 h, when VAN was administered every 12 h, approximately 7%–20% of these daily doses should be given additionally to obtain the desired AUC_24_/MIC and CFR targets. The appearance of subparallel shape for *D*_*van*_ versus *TBW* and of stepped shape for *D*_*van*_ versus *CL*_*cr*_, shown in Figure 4, implies that not *TBW* but *CL*_*cr*_ appears to have a significant association with *D*_*van*_, regardless of whether VAN is administered every 8 or 12 h.

Figure 3 shows the VAN-dose-graphical tool for pathogen populations or species with pooled MICs. It showed potential VAN dosing regimens at different *CL*_*cr*_, *TBW*, and pathogen species.

### Formulation Process of VAN Dosage Dynamic Adjustment

**FIGURE 5:**
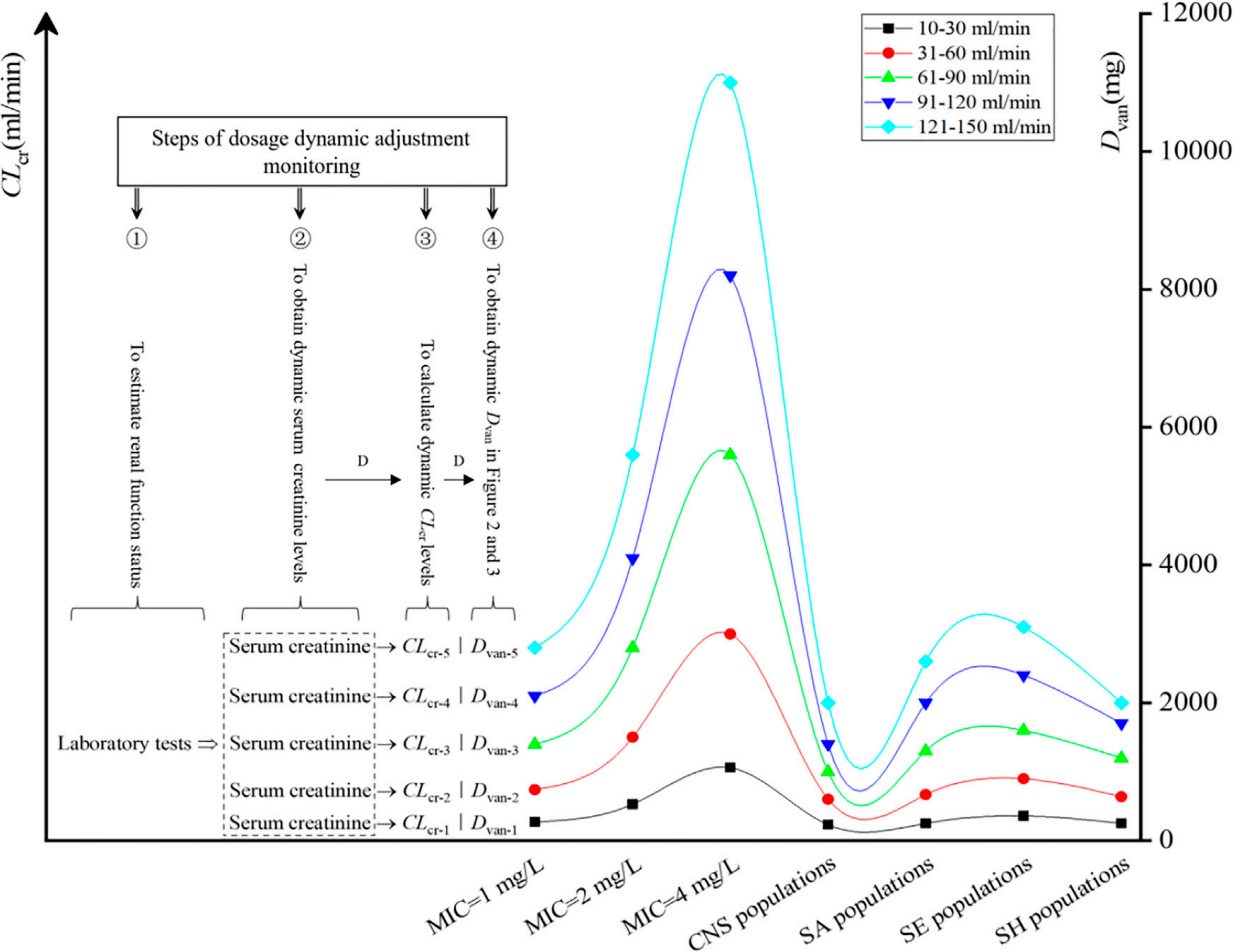
Steps of dosage dynamic adjustment monitoring. D, dynamics; CNS, coagulase-negative *Staphylococcus*; SA, *Staphylococcus aureus*; SE, *Staphylococcus epidermidis*; SH, *Staphylococcus haemolyticus*.

The formulation process of VAN dosage dynamic adjustment is shown in Figure 5. Four steps are included as follows: 1) estimating the renal function of the patient via laboratory tests; 2) obtaining dynamic serum creatinine levels and pathogen MIC or species; 3) calculating dynamic *CL*_cr_ levels by the Cockcroft–Gault method (Cockcroft and Gault, 1976); and 4) obtaining dynamic *D*_van_ in Figure 2 (for isolate with a definite MIC) or Figure 3 (for pathogen populations) to develop dynamic VAN dosing regimens. For details of formulation process of VAN dosage dynamic adjustment, see Supplementary Appendix S2: A short case report.

## Discussion

In the present study, we predicted the *D*_*van*_ using the established *D*_*van*_ model, constructed VAN-dose-graphical tools for the target patients suffering from both bacterial infection and hematological malignancies, and expounded the formulation process of VAN dosage dynamic adjustment. This study provided a very indicative, targeted, and specific reference for the formulation of VAN dosage regimens used in the simulated populations, and therefore we can readily know the required VAN dosage or regimens, regardless of whether VAN is used in empirical or follow-up therapy.

### The Derived AUC and *D*_*van*_ Model

Many studies (Moise et al., 2000; Jeffres et al., 2006; Neuner et al., 2010; Kullar et al., 2011; Holmes et al., 2013) that advocate a target VAN AUC for the AUC_24_/MIC ratio of 400 predict the AUC using a model established on *CL*_*van*_ and *CL*_*cr*_, i.e., AUC_24_ = dose per 24 h/[*CL*_*cr*_ × 0.79 + 15.4]×16 (also AUC_24_ = *D*_*van*_/*CL*_*van*_), which was derived from previous studies (Rodvold et al., 1988; Moise-Broder et al., 2004). In the estimation of VAN response, this application is also a consistent practice in most current studies (Moise-Broder et al., 2004; Jeffres et al., 2006; Holmes et al., 2013; Lewis, 2018). However, this simplified model for determining the AUC may cause deflected estimation, since 1) in this model, the impact of infusion time and rate on AUC_24_, which is quite important in the administration mode of using prolonged infusion, was not well considered. Of interest, VAN is just such an antibiotic that requires prolonged infusion. Understandably, ignoring the effect of these factors on AUC may result in an inaccurate AUC assessment; 2) this model is derived from the administration mode of using intravenous bolus and is therefore not well appropriate for antibiotics requiring prolonged infusion, such as VAN; and 3) the AUC_24_ determined by this model is measured based on a single dose and is referred to the total exposure from 0 h to infinity (i.e., AUC_∞_) (Rosenbaum, 2011; Drennan et al., 2019), in spite of a dose per 24 h used in this model. Understandably, this model is not well suitable for the situation of using multi-dose and intermittent administration. Thus, we derived the modified AUC_24_ model based on the classical PK formulas derived from the administration mode of using intravenous infusion and further obtained the *D*_van_ model. Conceivably, these models improve the predictability of outcomes.

### The Predicted VAN Dosage Requirements

The application of our results to clinical practice would consist of using Figures 2, 3 as nomograms to obtain, depending on the patient’s TBW and *CL*_cr_, the optimal VAN daily dosage for the treatment of infection due to the simulated *Staphylococcus* spp. These nomograms provide us with very useful directions on estimated VAN dosage regimens, regardless of whether VAN is used in empirical or follow-up therapy.

In empirical therapy, due to the unavailability of the pathogen species and susceptibility before VAN therapy, one useful approach for formulating VAN dosage regimens in MCSs could be to use the cumulative probability of achieving the target exposure against a causative pathogen population to screen the regimens. The nomograms inform us that at a CFR of 90% as the clinical acceptability, a standard VAN dosage of 2,000 mg/day is often sufficient for SH and CNS populations even in infected patients with *CL*_cr_ of up to 121–150 ml/min. This is also the case for the SA population, but for these patients, approximately 2,500–3,000 mg/day should be requisite if VAN is used to resist the SA population. However, for the SE population, the dosage of 2,000 mg/day is adequate only for patients with *CL*_cr_ of ≤90 ml/min. Understandably, this standard schedule seems preferable for the treatment of *Staphylococcus* spp. infection occurring in patients with *CL*_cr_ of ≤90 ml/min, implying that it should be questioned when used in those with *CL*_*cr*_ of >90 ml/min. Consistently, del Mar Ferna′ndez de Gatta et al. (2009) also questioned this standard schedule since very low CFRs were observed in patients with *CL*_*c*r_ of even >60 ml/min when this dosage was simulated against *Staphylococcus* spp. (a CFR of <60% for SA population, <40% for CNS population and SH population, and <30% for SE population).

In follow-up therapy, with availability of the bacterial culture and susceptibility test, one useful approach for formulating VAN dosage regimens in MCSs could be to use the PTA of achieving the target exposure against a causative isolate or a definite MIC. From these nomograms, it can be seen that at a PTA of 90% as the clinical acceptability, when VAN is used to resist *Staphylococcus* spp. isolates with an MIC of 1 mg/L and administered every 8 h, a dosage of approximately 270, 740, 1,400, 2,100, and 2,800 mg/day for patients with *CL*_cr_ of 10–30, 31–60, 61–90, 91–120, and 121–150 ml/min, respectively, may be required, regardless of the patient’s TBW. Interestingly, when VAN is used to resist *Staphylococcus* spp. isolates with an MIC of 2 and 4 mg/L, approximately twice and four times the aforementioned dosage, respectively, may be needed. Speculatively, a VAN dosage of standard 2,000 mg/day might be more suitable for the treatment of *Staphylococcus* spp. infection due to isolates with MICs of ≤1 mg/L and for patients with *CL*_cr_ of ≤90 ml/min, and a dosage of tolerable 4,000 mg/day might be more suitable for the treatment of *Staphylococcus* spp. infection due to isolates with MICs of ≤2 mg/L and for patients with *CL*_cr_ of ≤90 ml/min. Thus, a VAN dosage of 2,000 mg/day as a standard regimen for isolates with MICs of >1 mg/L might be questioned although part of these isolates, such as those with MICs of 1–4 mg/L, are currently considered susceptible to VAN.

Of interest, these nomograms also informed us that to achieve the desired AUC_24_/MIC and PTA or CFR targets, the *D*_van_ at the regimen of dosing every 12 h appeared to be significantly higher than that at the regimen of dosing every 8 h, regardless of the patient’s TBW and *CL*_*cr*_. It suggested that at the same VAN daily dose, the administration mode using multiple dosing may be more competitive. Furthermore, to achieve the desired AUC_24_/MIC target, the VAN dosage must vary with *CL*_*cr*_. This is very important for patients with acute renal impairment or recovery since fluctuant renal function, which is reflected by changed *CL*_*cr*_, will cause altered VAN exposure and resultant efficacy or nephrotoxicity. Fortunately, these charts provide useful directions on VAN dosage used at different renal function stages. In addition, for any one of the physiology-infection states consisting of *TBW*–*CL*_*cr*_–MIC or pathogen species, the nomograms afford the estimated VAN dosage. Combined with the formulation process of VAN dosage dynamic adjustment described in Figure 5, clinicians can easily formulate an optimal VAN dosage regimen for the target patients based on these factors, regardless of whether VAN is used in empirical or follow-up therapy.

Another interesting phenomenon is that a subparallel shape of *D*_*van*_ *vs. TBW* and a stepped shape of *D*_*van*_ *vs. CL*_*cr*_ were observed, as shown in Figure 4. It suggested that not *TBW* but *CL*_*cr*_ creates significant influence on the determination of *D*_*van*_, thus implying that a “one dose fits all *TBW*” approach seems feasible in the patients with hematological malignancies when they have relatively stable renal function. However, this dosing approach is inconsistent with the TBW-based dosing approach recommended in the 2020 VAN therapeutic guideline (Rybak et al., 2020b). However, this guideline does not offer specific dosage regimens or recommendations about VAN used in cancer patients (Rybak et al., 2020b).

From these PK/PD analyses, the need for dosage, tailored according to population kinetics (mainly *CL*_*cr*_), pathogen susceptibility (mainly MIC), and dosing strategy (mainly *τ*), seems evident. These considerations make it clear that the traditional “one dose fits all cases” approach to VAN, although logistically attractive, is grossly flawed. However, these optimal regimens based on the PK/PD strategy cannot replace a clinical study, and the possibility of VAN nephrotoxicity, especially at a high estimated dosage, is another important issue that should be noted before its use in the clinical setting (Jeffres et al., 2007; Ingram et al., 2008; Lodise et al., 2008). Therefore, we suggest the use of these initial regimens but followed by therapeutic drug monitoring, which is cost-effective in this population (de Gatta et al., 1996). Also, it could be useful to investigate nephrotoxicity associated with a higher VAN dosage.

As a tool to assist prescribers in constructing dosing regimens, the MCS method has been widely used in optimized antibiotic therapy. MCS-based feasibility for optimizing exposure to improve antimicrobial effectiveness has been expounded and applied in OPTAMA studies (Kuti and Nicolau, 2005), and MCS-indicated theoretical efficacy has been demonstrated by Eguchi et al. (2010) in an *in vitro* PD model study on meropenem against *P. aeruginosa*, in which *in vitro* viable cell counts of *P. aeruginosa* strain was used as a measure for *in vitro* bactericidal activity of meropenem. Thus, we believe that our approach is appropriate since VAN population PK models used were derived from the patients with hematological malignancies (Buelga et al., 2005), and PK variability was taken into account; the PD target was adopted from the 2020 VAN therapeutic guideline (Rybak et al., 2020b); the MIC values correspond to those reported in the ATLAS database (The Micron Group, 2022); and the emulation infusion parameters were derived from medication practice. Therefore, the results on VAN dosage could be applied if the patient and pathogen populations match those considered here. If this was not the case, the same methodological procedure could be followed, but the actual PK (relationship between *CL*_*van*_ and *CL*_*cr*_ and *V*_*d*_ and *TBW* due to patient variables) and PD modeling (MIC distribution) would have to be used. It should be pointed out that other factors in addition to the AUC_24_/MIC ratio have been reported as variables affecting the clinical outcome of patients treated with VAN, such as immunocompetence, which may even demand higher PK/PD targets (Schentag, 2001). Of note, this factor should be attached more importance in cancer patients because these populations often have altered immunocompetence due to their chemotherapy or biotherapy. Also, if clinical trials could define the targets for such patients, our methodological approach would still be valid. Also, local resistance data would improve the reliability of the predictions. Although this model analysis lacks sufficient power to detect clinical outcomes to some extent, our results provide further justification for prospective clinical trials aimed at evaluating the potential influence of a pharmacodynamically targeted VAN dosing schedule on the clinical outcomes of this population.

## Conclusion

Patients with hematological malignancies might manifest physiology that is unlikely to be encountered in general patients. Due to the distorted antibiotic PK profile, the standard VAN dosage of 2,000 mg/day used in this population might need to be reevaluated, especially for patients with high *CL*_cr_ and for isolates with high MIC. Based on the PK/PD endpoints, the data presented support that for treating infected patients with hematological malignancies; a VAN dosage of standard 2,000 mg/day might be more suitable for the treatment of *Staphylococcus* spp. infection due to isolates with MICs of ≤1 mg/L and for patients with *CL*_cr_ of ≤90 ml/min; and a dosage of tolerable 4,000 mg/day might be more suitable for the treatment of *Staphylococcus* spp. infection due to isolates with MICs of ≤2 mg/L and for patients with *CL*_cr_ of ≤90 ml/min. Nonetheless, large trials are needed to validate these regimens and their clinical implication, especially involving the balance of efficacy and nephrotoxicity at a high dose. Therefore, we suggest the use of these initial regimens but followed by therapeutic drug monitoring considering high VAN PK variability in such patients.

## Data Availability

The original contributions presented in the study are included in the article/Supplementary Material; further inquiries can be directed to the corresponding author.
