# Supplementary material for: Predicted Vancomycin Dosage Requirement in Patients With Hematological Malignancies and Dosage Dynamic Adjustment
Source: Front Pharmacol. 2022 Jun 6;13:890748. doi: 10.3389/fphar.2022.890748 (PMC9207402; doi:10.3389/fphar.2022.890748)
Supplement: Supplementary file 1 [file DataSheet2.pdf]

Appendix S2: A short case report

On Nov. 4th, 2021, a 52-year-old man weighing 61 kg, suffering from acute lymphoblastic leukemia, was diagnosed with severe open head trauma and spinal injury after slip in bathroom. The next day symptoms of reduced urine output and bacterial infection (fever and increased C-reactive protein, etc.) appeared. On the same day, the physician empirically performed the treatment of symptomatic precautions for reduced renal function [with a serum creatinine (S_{cr}) value of 258.5 μmol/L) and piperacillin/tazobactam (Tazocin, Pfizer Ltd, 4500 mg q 8h) for infection. On Nov. 7th, 2021, the reduced renal function was slightly improved (with a S_{cr} value of 232.2 μmol/L), however, the infection symptoms were not well improved and thus the physician suspected the pathogenic bacteria was methicillin-resistant *Staphylococcus aureus* (MRSA). On the same day, the physician empirically performed the treatment of VAN (Vancocin, VIANEX S.A.(PLANT C), 500 mg q 12h) for infection and the collection of blood samples for bacterial culture and susceptibility test. On Nov. 9th, 2021, the renal function continued to improve (with a S_{cr} value of 157.2 μmol/L), and the infection symptoms were relieved and the physician continued to perform the original VAN regimen (500 mg q 12h). On Nov. 11th, 2021, the renal function (with a S_{cr} value of 98.7 μmol/L) and infection symptoms were significantly improved, and the laboratory examinations reported that the pathogenic bacteria was MRSA, with a VAN MIC of 0.5 mg/L, and the physician continued to perform the original VAN regimen (500 mg q 12h). On Nov. 12th, 2021, the renal function appeared to be slightly deteriorated (with a S_{cr} value of 112.4 μmol/L), however, the infection symptoms continued to relieve and disappear. On Nov. 13th, 2021, the physician withdrew VAN treatment. Based on the theory presented in the current study, recommended dynamic dosage for VAN in different periods for this case were shown in Table 1 (suppl).

In this case, it should be noted that according to changes in S_{cr} of this subject during VAN therapy (see Figure 2 (suppl)), the outcome of increased S_{cr} on Nov. 12th, 2021, in addition to being associated with disease progression, may be resulted from the excessive VAN dosage in practice before Nov. 9th, 2021 (compared with the recommended dosage) as VAN itself can result in renal impairment, especially at overdose treatment. Therefore, it is important for clinicians to dynamically adjust VAN dosage according to the fluctuant renal function of the subject.

Table 1 (suppl) Recommended dynamic VAN dosage for this subject

	Nov. 7th, 2021	Nov. 9th, 2021	Nov. 11th, 2021	Nov. 12th, 2021
General data				
Therapy type	empirical therapy	empirical therapy	follow-up therapy	follow-up therapy
For pathogen	SA populations	SA populations	SA isolate (MIC=0.5 mg/L)	SA isolate (MIC=0.5 mg/L)
S_{cr}	232.2 μmol/L	157.2 μmol/L	98.7 μmol/L	112.4 μmol/L
CL_{cr}	28.3 ml/min	41.7 ml/min	66.5 ml/min	58.4 ml/min
Recommended regimens				
• 8-hour dosing regimen (if adopted)				
Recommended D_{van}	245.4 mg	664.5 mg	1360.1 mg	726.5 mg
Recommended regimen	81.8 mg q 8h	221.5 mg q 8h	453.4 mg q 8h	242.2 mg q 8h
• 12-hour dosing regimen (if adopted)				
Recommended D_{van}	282.7 mg	767.2 mg	1585.7 mg	836.9 mg
Recommended regimen	141.4 mg q 12h	383.6 mg q 12h	792.9 mg q 12h	418.5 mg q 12h

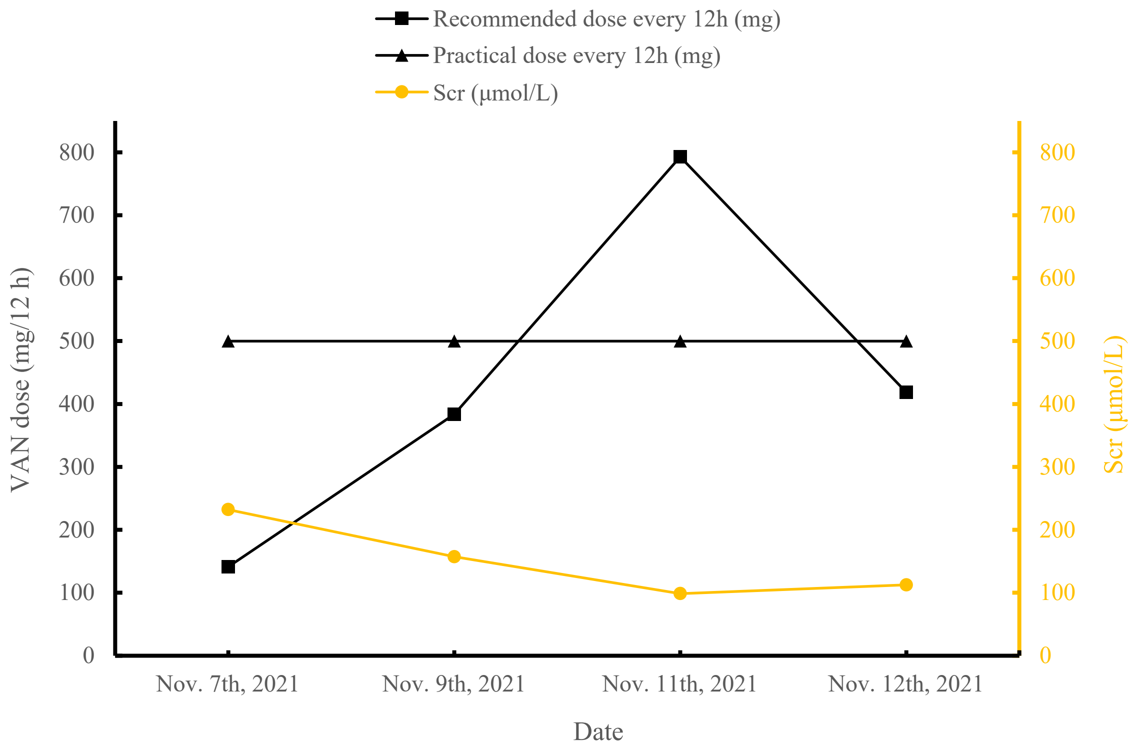

Figure 2 (suppl) Changes in S_{cr} of the subject during VAN therapy
