# Supplementary material for: Predicted Vancomycin Dosage Requirement in Patients With Hematological Malignancies and Dosage Dynamic Adjustment
Source: Front Pharmacol. 2022 Jun 6;13:890748. doi: 10.3389/fphar.2022.890748 (PMC9207402; doi:10.3389/fphar.2022.890748)
Supplement: Supplementary file 2 [file DataSheet1.pdf]

Appendix S1: Derivation of AUC_{24}/MIC

According to the book《Basic Pharmacokinetics and Pharmacodynamics: An Integrated Textbook and Computer Simulations》(the first edition edited by Rosenbaum SE and published by Wiley in 2011), in the administration mode of using intermittent infusion (see supplementary Figure 1), relationship of concentration (*C*) *v.s.* time (*t*) can be expressed by the following equations, assuming that the *in vivo* disposition of the target drug fits one-compartment model.

① during infusion (or in ascending branch of curve)

$$C = \frac{v}{KV_d} \cdot \left(1 - e^{-Kt}\right)\left(0 \le t \le t_{\text{inf}}\right)$$

Obviously, C achieves the maximum (C_{max}) at t_{inf}.

② after completion of infusion (or in descending branch of curve)

$$C' = C_{\max} \cdot e^{-Kt}\left(0 \le t < \infty\right)$$

Set $v/(KV_d)$ as A, then,

$$C = A\left(1 - e^{-Kt}\right)$$

$$C_{\max} = A\left(1 - e^{-Kt_{\text{inf}}}\right)$$

Set $A(1-e^{-K\,t_{inf}})$ as B, then,

$$C' = Be^{-Kt}$$

Note: C, concentration in ascending branch of curve; C', concentration in descending branch of curve; K, elimination rate constant; V_d, distribution volume; t, time during or after infusion; t_{inf}, infusion time; v, zero-order infusion rate, calculated as each dose divided by infusion time [i.e., D_{van}/(24/dosing interval "τ")]/t_{inf}; C_{max}, maximum concentration; e, natural constant.

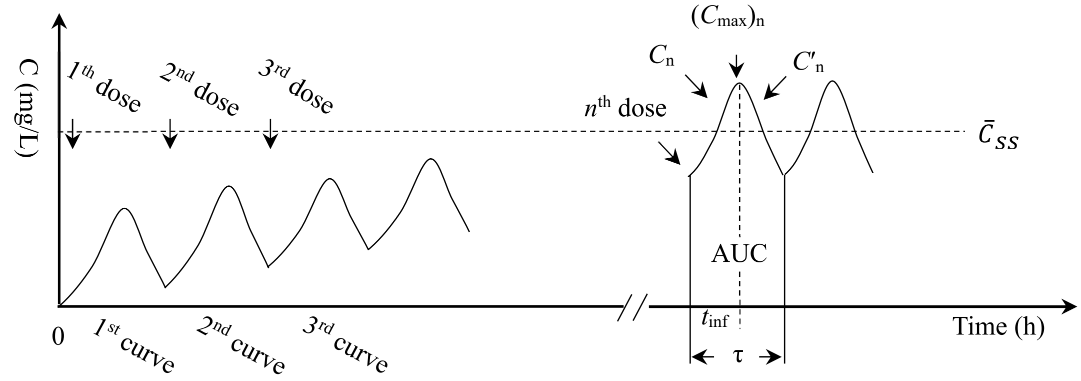

Figure 1 (Suppl) *C*-*t* curve in the administration mode of using intermittent infusion. t_{inf}, infusion time; τ, dosing interval; C_n, concentration in ascending branch of nth curve; $(C_{max})_n$, maximum concentration of nth curve; C'_n, concentration in descending branch of nth curve; $\bar{C}_{ss}$, mean steady-state concentration; AUC, area under curve.

From Figure 1 (Suppl), it can be seen that in any (set as "n^{th}") dose, C_n achieves $(C_{max})_n$ at t_{inf} and C'_n achieves $(C_{min})_n$ at τ. Based on the above equations, it can be seen that in 1st dose, $(C_{max})_1$ is equal to B and $(C_{min})_1$ is equal to $Be^{-K(\tau-t_{inf})}$; and in 2nd dose, $(C_{max})_2$ will be equal to $[(C_{max})_1+(C_{min})_1]$ due to the residual concentration of $(C_{min})_1$ in 1st dose, and $(C_{min})_2$ will be equal to $(C_{max})_2\cdot e^{-K(\tau-t_{inf})}$. By analogy, C_n and C'_n in n^{th} dose can be deduced, as well as $(C_{max})_n$ and $(C_{min})_n$. Derivation of C_n, C'_n, $(C_{max})_n$ and $(C_{min})_n$ in any (n^{th}) dose is as follows:

(1) in 1st dose (or 1st curve)

- $C_1 = A(1-e^{-Kt})$
- $(C_{max})_1 = B$
- $C'_1 = Be^{-Kt'}$
- $(C_{min})_1 = Be^{-K(\tau-t_{inf})}$

(2) in 2nd dose (or 2nd curve)

- $C_2 = Be^{-K(\tau-t_{inf})}+A(1-e^{-Kt})$
- $(C_{max})_2 = Be^{-K(\tau-t_{inf})}+A(1-e^{-K\,t_{inf}}) = B[1+e^{-K(\tau-t_{inf})}]$
- $C'_2 = B[1+e^{-K(\tau-t_{inf})}]e^{-Kt'}$
- $(C_{min})_2 = B[1+e^{-K(\tau-t_{inf})}]e^{-K(\tau-t_{inf})} = B[e^{-K(\tau-t_{inf})}+e^{-2K(\tau-t_{inf})}]$

(3) in 3rd dose (or 3rd curve)

- $C_3 = B[e^{-K(\tau-t_{inf})}+e^{-2K(\tau-t_{inf})}]+A(1-e^{-Kt})$
- $(C_{max})_3 = B[e^{-K(\tau-t_{inf})}+e^{-2K(\tau-t_{inf})}]+A(1-e^{-K\,t_{inf}}) = B[1+e^{-K(\tau-t_{inf})}+e^{-2K(\tau-t_{inf})}]$
- $C'_3 = B[1+e^{-K(\tau-t_{inf})}+e^{-2K(\tau-t_{inf})}]e^{-Kt'}$
- $(C_{min})_3 = B[1+e^{-K(\tau-t_{inf})}+e^{-2K(\tau-t_{inf})}]e^{-K(\tau-t_{inf})} = B[e^{-K(\tau-t_{inf})}+e^{-2K(\tau-t_{inf})}+e^{-3K(\tau-t_{inf})}]$

By analogy,

(4) in nth dose (or nth curve)

- $C_n = B[e^{-K(\tau-t_{inf})}+e^{-2K(\tau-t_{inf})}+e^{-3K(\tau-t_{inf})}\cdots\cdots+e^{-(n-1)K(\tau-t_{inf})}]+A(1-e^{-Kt})$
- $(C_{max})_n = B[e^{-K(\tau-t_{inf})}+e^{-2K(\tau-t_{inf})}+e^{-3K(\tau-t_{inf})}\cdots\cdots+e^{-(n-1)K(\tau-t_{inf})}]+A(1-e^{-K\,t_{inf}}) = B[1+e^{-K(\tau-t_{inf})}+e^{-2K(\tau-t_{inf})}+e^{-3K(\tau-t_{inf})}\cdots\cdots+e^{-(n-1)K(\tau-t_{inf})}]$
- $C'_n = B[1+e^{-K(\tau-t_{inf})}+e^{-2K(\tau-t_{inf})}+e^{-3K(\tau-t_{inf})}\cdots\cdots+e^{-(n-1)K(\tau-t_{inf})}]e^{-Kt'}$
- $(C_{min})_n = B[1+e^{-K(\tau-t_{inf})}+e^{-2K(\tau-t_{inf})}+e^{-3K(\tau-t_{inf})}\cdots\cdots+e^{-(n-1)K(\tau-t_{inf})}]e^{-K(\tau-t_{inf})} = B[e^{-K(\tau-t_{inf})}+e^{-2K(\tau-t_{inf})}+e^{-3K(\tau-t_{inf})}\cdots\cdots+e^{-(n-1)K(\tau-t_{inf})}+e^{-nK(\tau-t_{inf})}]$

Set as,

$r = 1+e^{-K(\tau-t_{inf})}+e^{-2K(\tau-t_{inf})}+e^{-3K(\tau-t_{inf})}\cdots\cdots+e^{-(n-1)K(\tau-t_{inf})}$

Then,

$re^{-K(\tau-t_{inf})} = [e^{-K(\tau-t_{inf})}+e^{-2K(\tau-t_{inf})}+e^{-3K(\tau-t_{inf})}\cdots\cdots+e^{-(n-1)K(\tau-t_{inf})}+e^{-nK(\tau-t_{inf})}]$

By solving this equation, then,

$r = [1-e^{-nK(\tau-t_{inf})}]/[1-e^{-K(\tau-t_{inf})}]$

Thus,

(5) In nth dose (or nth curve)

- $C_n = B[e^{-K(\tau-t_{inf})}-e^{-nK(\tau-t_{inf})}]/[1-e^{-K(\tau-t_{inf})}]+A(1-e^{-Kt})$
- $(C_{max})_n = B[1-e^{-nK(\tau-t_{inf})}]/[1-e^{-K(\tau-t_{inf})}]$
- $C'_n = B[1-e^{-nK(\tau-t_{inf})}]/[1-e^{-K(\tau-t_{inf})}]e^{-Kt'}$
- $(C_{min})_n = B[1-e^{-nK(\tau-t_{inf})}]/[1-e^{-K(\tau-t_{inf})}]e^{-K(\tau-t_{inf})}$

When n is close to "infinity" or reaches the steady state, $e^{-nK(\tau-t_{inf})}$ is close to "0". Then,

(6) In nth dose (or nth curve)

- $C_n = Be^{-K(\tau-t_{inf})}/\ [1-e^{-K(\tau-t_{inf})}]+A(1-e^{-Kt})$
- $C_{max} = Be^{-K(\tau-t_{inf})}/\ [1-e^{-K(\tau-t_{inf})}]+A(1-e^{-Kt_{inf}})$
- $C'_n = \{B/[1-e^{-K(\tau-t_{inf})}]\}e^{-Kt'}$
- $(C_{min})_n = \{B/[1-e^{-K(\tau-t_{inf})}]\}e^{-K(\tau-t_{inf})}$

According to Figure 1 (Suppl) and the integral relationship for AUC calculation, then,

$\text{AUC} = \int C_n\,dt + \int C'_n\,dt$

According to the definite integration rule,

$$\text{AUC} = \int_0^{t_{inf}} \left[Be^{-K(\tau-t_{inf})}/(1-e^{-K(\tau-t_{inf})}) + A\left(1-e^{-Kt}\right)\right]dt + \int_0^{\tau-t_{inf}} [B/(1-e^{-K(\tau-t_{inf})})]e^{-Kt}dt$$

Due to the following relationship in one-compartment model,

$CL = KV_d$

$B = A(1-e^{-K\,t_{inf}})$

$A = v/(KV_d)$

And combining the calculation rules of definite integral, then,

$$\text{AUC} = \frac{v\cdot t_{inf}}{CL}\cdot\frac{1-e^{-CL/V_d\cdot\tau}}{1-e^{-CL/V_d\cdot(\tau-t_{inf})}} + \frac{v\cdot\left(e^{-CL/V_d\cdot t_{inf}}-1\right)}{V_d}\cdot\frac{e^{-CL/V_d\cdot(\tau-2t_{inf})}-e^{-CL/V_d\cdot(\tau-t_{inf})}}{1-e^{-CL/V_d\cdot(\tau-t_{inf})}}$$

Due to 24/τ curves being formed within 24 h, then,

$$\mathrm{AUC}_{24} = \frac{24}{\tau} \cdot \left[\frac{v \cdot t_{\inf}}{CL} \cdot \frac{1 - e^{-CL/V_d \cdot \tau}}{1 - e^{-CL/V_d \cdot (\tau - t_{\inf})}} + \frac{v \cdot \left(e^{-CL/V_d \cdot t_{\inf}} - 1 \right)}{V_d} \cdot \frac{e^{-CL/V_d \cdot (\tau - 2t_{\inf})} - e^{-CL/V_d \cdot (\tau - t_{\inf})}}{1 - e^{-CL/V_d \cdot (\tau - t_{\inf})}} \right]$$

By transformation, then,

$$\mathrm{AUC}_{24} = \frac{24v}{\tau \cdot \left(e^{CL/V_d \cdot \tau} - e^{CL/V_d \cdot t_{\inf}} \right)} \left[\frac{t_{\inf} \left(e^{CL/V_d \cdot \tau} - 1 \right)}{CL} - \frac{\left(e^{CL/V_d \cdot t_{\inf}} - 1 \right)^2}{V_d} \right]$$

Thus,

$$\mathrm{AUC}_{24} / \mathrm{MIC} = \frac{\frac{24v}{\tau \cdot \left(e^{CL/V_d \cdot \tau} - e^{CL/V_d \cdot t_{\inf}} \right)} \left[\frac{t_{\inf} \left(e^{CL/V_d \cdot \tau} - 1 \right)}{CL} - \frac{\left(e^{CL/V_d \cdot t_{\inf}} - 1 \right)^2}{V_d} \right]}{\mathrm{MIC}}$$
